# Therapeutic effect of Sheng Mai San, a traditional Chinese medicine formula, on inflammatory bowel disease via inhibition of NF-κB and NLRP3 inflammasome signaling

**DOI:** 10.3389/fphar.2024.1426803

**Published:** 2024-08-02

**Authors:** Magnolia Muk-Lan Lee, Brandon Dow Chan, Yuen-Wa Ng, Tsz-Wing Leung, Tan-Yu Shum, Jian-Shu Lou, Wing-Yan Wong, William Chi-Shing Tai

**Affiliations:** ^1^ Department of Applied Biology and Chemical Technology, The Hong Kong Polytechnic University, Hung Hom, Hong Kong SAR, China; ^2^ The Laboratory for Probiotic and Prebiotic Research in Human Health, The Hong Kong Polytechnic University, Hung Hom, Hong Kong SAR, China; ^3^ School of Pharmacy, Hangzhou Normal University, Hangzhou, Zhejiang, China; ^4^ State Key Laboratory of Chinese Medicine and Molecular Pharmacology (Incubation), Shenzhen Research Institute, The Hong Kong Polytechnic University, Shenzhen, China

**Keywords:** inflammatory bowel disease, Sheng Mai San, inflammasome, macrophages, NF-κB, NLRP3 inflammasome

## Abstract

**Introduction:**

Inflammatory bowel disease (IBD) is a globally emergent chronic inflammatory disease which commonly requires lifelong care. To date, there remains a pressing need for the discovery of novel anti-inflammatory therapeutic agents against this disease. Sheng Mai San (SMS) is a traditional Chinese medicine prescription with a long history of use for treating *Qi* and *Yin* deficiency and recent studies have shown that SMS exhibits anti-inflammatory potential. However, the effects of SMS on the gastrointestinal system remain poorly studied, and its therapeutic potential and underlying molecular mechanisms in IBD have yet to be discovered. In this study, we examined the therapeutic efficacy of SMS in IBD and its anti-inflammatory activity and underlying molecular mechanism, *in vivo* and *in vitro*.

**Methods:**

The therapeutic efficacy of SMS in IBD was assessed in the DSS-induced acute colitis mouse model. Body weight, stool consistency, rectal bleeding, colon length, organ coefficient, cytokine levels in colon tissues, infiltration of immune cells, and colon pathology were evaluated. The anti-inflammatory activity of SMS and related molecular mechanisms were further examined in lipopolysaccharide (LPS)-induced macrophages via assessment of pro-inflammatory cytokine secretion and NF-κB, MAPK, STAT3, and NLRP3 signalling.

**Results:**

SMS significantly ameliorated the severity of disease in acute colitis mice, as evidenced by an improvement in disease activity index, colon morphology, and histological damage. Additionally, SMS reduced pro-inflammatory cytokine production and infiltration of immune cells in colon tissues. Furthermore, in LPS-induced macrophages, we demonstrated that SMS significantly inhibited the production of cytokines and suppressed the activation of multiple pro-inflammatory signalling pathways, including NF-κB, MAPK, and STAT3. SMS also abolished NLRP3 inflammasome activation and inhibited subsequent caspase-1 activation and IL-1β secretion, suggesting a new therapeutic target for the treatment of IBD. These mechanistic findings were also confirmed in *in vivo* assays.

**Conclusion:**

This study presents the anti-inflammatory activity and detailed molecular mechanism of SMS, *in vitro* and *in vivo*. Importantly, we highlight for the first time the potential of SMS as an effective therapeutic agent against IBD.

## 1 Introduction

Inflammatory bowel disease (IBD), including ulcerative colitis and Crohn’s disease, is an emerging chronic and recurrent inflammatory disease of the intestines. IBD can lead to a substantial decline in the quality of life through symptoms such as abdominal pain, diarrhoea, or weight loss (Knowles et al., 2018; Calviño-Suárez et al., 2021).

Research suggests that a dysregulated immune response is a key contributor to the aberrant inflammation observed in the intestines of IBD patients. Specifically, macrophages play a crucial role in the maintenance and regulation of intestinal homeostasis (Na et al., 2019; Dharmasiri et al., 2021). In the pathogenesis of IBD, homeostasis of the intestine is disrupted due to an aberrant immune response directed against gut bacteria. Infiltration and activation of macrophages in the mucosal layer leads to oxidative stress, proteolytic damage, and generation of inflammatory mediators and cytokines including NO, IL-6, and IL-1β, through activation of inflammatory signaling pathways such as NF-κB, MAPK, and STAT3. Upon release, these cytokines can bind and activate receptors on immune cells and trigger the adaptive immune response to further generate inflammatory mediators and cytokines, in a positive feedback loop (Zhou et al., 2019).

Currently, there is no cure for IBD, and commonly used therapeutics such as 5-aminosalicylic acid (5-ASA), corticosteroids, antibiotics, and monoclonal antibodies are hindered by limited effectiveness and side effects (Dinarello, 2010; Sandborn, 2016).

Sheng Mai San (SMS) is a traditional Chinese medicine (TCM) prescription, composed of *Ginseng Radix et Rhizoma*, *Ophiopogonis Radix*, and *Schisandrae chinensis* Fructus. SMS was first documented in “Yixue Qiyuan” in the 12th century and has been widely used for treating *Qi* and *Yin* deficiency, restoring pulse, and arresting excessive perspiration based on TCM theory (Cao et al., 2016). Due to similarities in clinical manifestation of *Qi* and *Yin* deficiency syndrome and the major complications of heat-stroke (e.g., ischemic brain injury, myocardial and respiratory dysfunction), SMS has been used to treat respiratory, cardiovascular, and cerebrovascular diseases. While the traditional use of SMS has centred around the aforementioned diseases, a common outcome observed amongst the diseases is a pro-inflammatory response. In previous studies, SMS has demonstrated inhibition of pro-inflammatory signaling molecules in renal ischemia and heat stress animal models (Lee et al., 2005; Zhang X. et al., 2022), as well as suppression of pro-inflammatory signaling in acute lung injury rats (Zhang F. H. et al., 2022) and zebrafish (Zheng et al., 2021), suggesting its anti-inflammatory potential. However, to date, the effects of SMS in gastrointestinal inflammatory disease have not been investigated, and the therapeutic potential and molecular mechanisms of SMS in IBD have yet to be discovered.

Therefore, the aim of this study was to investigate the potential anti-inflammatory properties of SMS and the mechanisms involved, as well as its potential as a therapeutic against IBD, *in vitro* and *in vivo*. Our findings demonstrate that SMS significantly reduced disease severity, ameliorated colonic damage, and decreased intestinal inflammation in dextran sulfate sodium (DSS)-induced acute colitis mice. Through further *in vitro* mechanistic studies in lipopolysaccharide (LPS)-induced macrophages, we demonstrated that SMS significantly inhibited pro-inflammatory mediator production and suppressed the activation of pro-inflammatory signalling pathways including NF-κB, STAT3, MAPK, and the NLRP3 inflammasome. Altogether, our results suggest the potential of SMS as an effective therapeutic against IBD.

## 2 Materials and methods

### 2.1 SMS

Concentrated powder of SMS (batch: A210005110), comprising the three Chinese herbal medicines *Ginseng Radix et Rhizoma*, *Ophiopogonis Radix*, and *Schisandrae chinensis* Fructus (1.7:2.5:1), was purchased from Nong’s Company Limited (Hong Kong, China).

### 2.2 LC-MS/MS analysis

Chemical standards, including ginsenoside Rg1 (Rg1) (purity ≥ 98%), ophiopogonin D (OP) (purity ≥ 98%), schisandrol A (SA) (purity ≥ 98%), and schizandrin B (SB) (purity ≥ 98%) were purchased from Chengdu Must Bio-technology Co., Ltd. (Chengdu, China). Digoxin (purity ≥95%, Sigma-Aldrich, United States) was run in parallel as an internal standard.

Calibration standards were prepared to 10, 20, 50, 100, 200, 500, and 1000 μg/L in 50% methanol and spiked with 50 µL internal standard (1000 ng/mL digoxin). Standards were mixed thoroughly and centrifuged at 13,000× g for 10 min at 4°C. Supernatants were collected to a liquid chromatography vial for liquid chromatography-tandem mass spectrometry (LC-MS/MS) analysis. The injection volume was 3 µL.

Precisely 0.2 g SMS extract was weighed and spiked with 100 µL of the internal standard (1000 μg/L). The sample was then dissolved in 900 µL of water and sonicated for 10 min at room temperature. Next, 500 µL of the sample was combined with 500 µL methanol and vortexed for 30 s. The mixture was then centrifuged at 13,000× g for 10 min at 4°C, and the supernatant collected for LC-MS/MS analysis. The injection volume for the analysis was 3 µL.

The selected chemical markers were quantified using an Agilent 1290 LC and Agilent 6460 Electrospray Ionization Triple Quadrupole Mass Spectrometer (LC-ESI-QQQ MS/MS). For SA, SB, and OP, the LC separation was carried out with an ACQUITY UPLC BEH C18 column (1.7 µm particle size, 2.1 × 50 mm) (Waters, Manchester, United Kingdom) and an ACQUITY UPLC BEH C18 VanGuard pre-column (Waters, Manchester, United Kingdom). The mobile phase used a solvent system of 0.1% formic acid (purity ≥ 95%, Sigma-Aldrich) in water as solvent A, and 0.1% formic acid in acetonitrile (LC-MS grade, Duksan Pure Chemical, Korea) as solvent B. Separation was performed under gradient elution as follows: 5% B at 0–1 min, 20%–80% B at 1–20 min, 80%–95% B at 20–25 min, 95% B at 25–26 min, 5% B at 26–30 min. The flow rate was 0.3 mL/min. The column temperature was set to 40°C. The separation of Rg1 was carried out with an ACQUITY UPLC Protein BEH C4 (1.7 µm particle size, 2.1 × 100 mm) column (Waters) and an ACQUITY UPLC Protein BEH C4 VanGuard Pre-column (Waters). The mobile phase was as mentioned above. Separation was performed under gradient elution as follows: 5% B at 0–1 min, 5%–95% B at 1–13 min, 95% B at 13–14 min, 95%–5% B at 14–14.1 min, 5% B at 14.1–15 min. The flow rate was 0.4 mL/min. The column temperature was set to 40°C; The conditions of the ESI source were as follows: Temperature 300°C; Gas flow 8 L/min; Sheath gas 11 L/min; Delta EMV(+) 600 V; Delta EMV(−) 500 V. The MRM settings of individual chemical markers are as indicated in Table 1.

**TABLE 1:** MRM settings for MS analysis.

Chemical marker	Mode	Precursor ion (m/z)	Product ion (m/z)	Fragmentor (V)	Collision energy (V)
Ginsenoside Rg1	Negative	845.4	799.4	170	20
Schisandrol A	Positive	455.2	409.1	180	20
Schizandrin B	Positive	423.2	377.0	180	20
Ophiopogonin D	Positive	877.4	447.0	200	55
Digoxin	Positive	969.1	789.1	200	55
Digoxin	Negative	945.5	799.4	150	20

### 2.3 Anti-inflammatory effect

#### 2.3.1 *In vitro* assessment

##### 2.3.1.1 Cell lines

RAW264.7 and J774A.1 murine macrophages, purchased from the American Type Culture Collection (Manassas, United States), were maintained in Dulbecco’s Modified Eagle Medium (DMEM) (Life Technologies, United States) supplemented with 10% heat-inactivated Fetal Bovine Serum (FBS) and penicillin/streptomycin (50 U/mL) at 37°C, 5% CO_2_. J774 Dual murine macrophages (reporter cell line) were purchased from Invivogen (San Diego, CA, United States) and cultured in DMEM supplemented with 10% heat-inactivated FBS, 5 μg/mL Blasticidin, 100 μg/mL Zeocin and 100 μg/mL Normocin. Cell lines were tested and confirmed to be free of *mycoplasma* contamination.

##### 2.3.1.2 Cell viability assay

The viability of RAW264.7 and J774A.1 macrophages treated with SMS were assessed using the 3-[4,5-dimethylthiazol-2-yl]-2,5-diphenyltetrazoliumbromide (MTT) assay. Briefly, cells were plated in a 96-well plate at a density of 1 × 10^4^ cells at 37°C overnight. SMS powder was freshly prepared in cell culture medium and filtered with a 0.2 µm syringe filter. Cells were treated with SMS with or without 1 μg/mL LPS (Sigma-Aldrich) for 24 h followed by incubation with 20 μL MTT (2.5 mg/mL, Sigma-Aldrich) for 4 h. Media was removed after incubation and DMSO (Duksan) was added to each well to dissolve formazan crystals. Absorbance at 570 nm was measured in each well using a Varioskan LUX Multimode Microplate Reader (Thermo Scientific, United States).

##### 2.3.1.3 Measurement of NO levels

NO levels in conditioned media from macrophages were measured using the Griess Reagent System (Promega, Madison, WI, United States). Briefly, cells were pre-treated with different concentrations of SMS for 3 h followed by stimulation with 1 μg/mL LPS for 24 h. Thereafter, NO concentration in conditioned media was quantified according to manufacturer’s instructions.

##### 2.3.1.4 Enzyme-linked immunosorbent assay (ELISA)

IL-6, IL-1β, TNF-α, and IL-10 levels in conditioned media or colon cultures were measured using ELISA kits (BioLegend, Cambridge, United Kingdom) according to manufacturer’s instructions.

##### 2.3.1.5 Immunofluorescence staining of NF-κB p65

1 × 10^5^ RAW264.7 macrophages were seeded in a μ-Slide eight well chambered coverslip (ibidi, Martinsried, Germany) overnight. Cells were starved for 2 h in serum free medium, followed by pre-treatment with or without SMS for 3 h before co-treatment with 1 μg/mL LPS for 15 min. Cells were then fixed, permeabilized, and stained for NF-κB. Nuclei were stained with 300 nM DAPI solution (Molecular Probes, Life Technologies) and then mounted with fluorescence mounting medium (Dako, Agilent, Santa Clara, CA, United States). The nuclear translocation of NF-κB was examined by fluorescence microscopy using a Leica TCS SPE Confocal Microscope (Leica Microsystems, Wetzlar, Germany).

##### 2.3.1.6 qPCR

Total RNA was isolated from murine cells and colon samples using the E.Z.N.A.^®^ Total RNA Kit I (Omega Bio-tek, United States), and quantified using a Nanodrop One spectrophotometer (Thermo Scientific). First strand cDNA synthesis was carried out from 1 μg RNA using SuperScript^®^ VILO™ MasterMix (Thermo Scientific). PCR reaction mixtures contained 10 μL of 2× SYBR Green Master Mix (Applied Biosystems, United States), 10 μM of forward and reverse primers, and 1 μL sample cDNA. Amplification was performed using the QuantStudio7 system (Applied Biosystems) at the following conditions: 2 min at 50°C, 2 min at 95°C, followed by 40 cycles of 15 s at 95°C, and 1 min at 60°C. Relative gene expression was calculated using the 2^−ΔΔCT^ method with normalization to the expression level of β-actin. Primers used are listed in Supplementary Table S1.

##### 2.3.1.7 NF-κB reporter cell assay

J774-Dual cells were used to study the activation of the NF-κB pathway by assessing the activity of secreted embryonic alkaline phosphatase (SEAP). Briefly, cells were pre-treated with SMS for 3 h followed by stimulation with 1 μg/mL LPS for 24 h. After treatment, conditioned media were collected and used to measure SEAP production as induced by NF-κB activation, using QUANTI-Blue detection reagents (InvivoGen) according to manufacturer’s protocols.

##### 2.3.1.8 Induction of the NLRP3 inflammasome

J774A.1 macrophages were seeded in each well of a 6-well plate at a density of 1 × 10^6^ cells at 37°C overnight. Cells were then pre-treated with or without SMS for 3 h, stimulated with 1 μg/mL LPS for 4 h, and subsequently treated with 3 mM ATP or 20 µM nigericin for 30 min. Expression levels of NLRP3 and other related proteins in cell lysates and conditioned media were detected by Western blot. IL-1β levels in media were examined using ELISA; mRNA expression of NLRP3 was quantified using qPCR.

##### 2.3.1.9 Western blotting

Western blotting was carried out as previously reported (Wong et al., 2016). In brief, macrophages were harvested after SMS treatments. In some experiments, cells were fractionated into nuclear and cytoplasmic fractions using NE-PER™ Nuclear and Cytoplasmic Extraction Reagent kit (Thermo Scientific) according to the manufacturer’s instructions. Samples were lysed in RIPA buffer (50 mM Tris-HCl, pH7.4, 150 mM NaCl, 1 mM EDTA, 1% Triton X-100, 1% sodium deoxycholate, 0.1% SDS) and the protein concentrations of the lysates were determined using DC Protein Assay (Bio-Rad, Hercules, CA, United States). Equal amounts of lysates were electrophoresed through SDS-PAGE gels and transferred onto PVDF membranes (Bio-Rad). The blots were then blocked in 5% non-fat skim milk and probed with diluted primary antibodies overnight: iNOS and COX2 (Cayman Chemical Company, United States), phospho-IKKα/β, IKKα, IKKβ, phospho-NF-κB, NF-κB, phospho-IKBα, IKBα, phospho-ERK, ERK, phospho-p38, p38, phospho-JNK, JNK, phospho-STAT3, STAT3, Cleaved caspase-1, Cleaved IL-1β, and IL-1β (Cell Signaling Technology, United States), NLRP3 (AdipoGen Life Sciences, San Diego, CA), Histone H1, GAPDH, ASC, Caspase-1, and β-actin (Santa Cruz Biotechnology, United States). Blots were then incubated with corresponding goat anti-rabbit or goat anti-mouse (Life Technologies) HRP-conjugated secondary antibodies. Protein bands were visualized using Clarity ECL or Clarity Max Western blotting substrates (Bio-Rad). Images were obtained using a ChemiDoc Imaging System (Bio-Rad) and protein expression was analyzed using Image Lab software (Bio-Rad).

#### 2.3.2 *In vivo* assessment

##### 2.3.2.1 Animals

6–8 week-old male and female wildtype C57BL/6J mice, weighing 18–22 g, were purchased from the Jackson Laboratory (Bar Harbor, ME, United States) and were mated to maintain an inbred breeding colony at the Centralized Animal Facilities of The Hong Kong Polytechnic University. Throughout the acclimatization and study periods, mice were kept in a barrier-sustained animal house, air-conditioned at 20°C ± 2°C and humidity maintained at 55% ± 10%, under a 12-h light/dark cycle. Food and water were available *ad libitum*. All animal experiments were approved by the Hong Kong Polytechnic University Animal Subjects Ethics Sub-committee (ASESC) and conducted in accordance with the Institutional Guidelines and Animal Ordinance of the Department of Health, H.K.S.A.R.

##### 2.3.2.2 Peritoneal macrophages

Peritoneal macrophages were collected from 8 to 10 week-old male C57BL/6J mice induced with thioglycolate. Briefly, mice were injected intraperitoneally with 3% thioglycolate and induced for 3 days. At the end of the induction period, mice were sacrificed by cervical dislocation and peritoneal macrophages were collected by rinsing the peritoneal cavity with 5 mL PBS twice. Cell suspensions were combined and centrifuged at 300× g for 10 min at 4°C. Red blood cells in the cell pellet were lysed using 0.5 mL ACK lysing buffer (Thermo Scientific) for 1 min at room temperature, and 5 mL PBS was then added. After centrifugation at 300× g for 10 min at 4°C, cells were resuspended in RPMI1640 (Life Technologies) supplemented with 10% heat-inactivated FBS and penicillin/streptomycin (50 U/mL), and seeded in a 60 mm plate at a density of 4 × 10^6^ cells at 37°C for 2 h. After incubation, culture media was removed and replaced with RPMI1640 supplemented with 50 μM 2-mercaptoethanol (Sigma-Aldrich). Cells were then cultured overnight and treated as mentioned above.

##### 2.3.2.3 DSS-induced colitis mouse model and treatments

The acute DSS-induced colitis mouse model was established as previously described, with minor modifications (Wong et al., 2017). 8–10 week-old male C57BL/6J mice were randomly assigned to five groups (n = 6 per group), colitis was induced in four of the groups by providing mice with 2.5% w/v DSS (reagent grade; 36,000–50,000Da; MP Biomedicals, Solon, OH, United States) in their drinking water for seven consecutive days. SMS powder was freshly dissolved in Milli Q water. Mice were treated daily by oral gavage as follows: (1) vehicle: Milli Q water; (2) low dose SMS (SMS-LD): 2 g/kg SMS in Milli Q water; (3) high dose SMS (SMS-HD): 4 g/kg SMS in Milli Q water; (4) 5-ASA: 200 mg/kg in 0.5% CMC. 5-ASA was chosen as a positive control as it is one of the most commonly used first-line drugs for the treatment of IBD (Raine et al., 2022). A control group received drinking water (without DSS) with vehicle treatment. An illustrated outline of the experiment is presented in Supplementary Figure S1. Doses of SMS and 5-ASA were chosen based on translating commonly used human doses (SMS: 10 g/d, 5-ASA: 1 g/d) to mouse equivalent doses, as previously described (Reagan-Shaw et al., 2008).

##### 2.3.2.4 Assessment of colitis

During the experiment period, mice body weight, food and water consumption were measured daily. The degree of colitis was evaluated via disease activity index (DAI), which was calculated as previously described by Marín et al. (Marín et al., 2013). The DAI scoring system is the sum of scores for weight loss, stool consistency, and visible blood in feces which was also scored daily. At the end of the experiment, mice were sacrificed, intestines were removed, and colon lengths were measured. Colons were opened longitudinally and washed with saline. Colon sections were either fixed in formalin solution for histopathological assessment, cultured in medium for cytokine level assessment, or snap frozen at −80°C for protein analysis.

##### 2.3.2.5 Colon tissue culture

Culture of colon tissues was performed as described by Wirtz et al. (Wirtz et al., 2007), with slight modifications. In brief, approximately 1 cm sections of mouse colons were isolated, washed thoroughly with sterile PBS, and cultured in 1 mL RMPI 1640 culture medium supplemented with 10% FBS at 37°C for 24 h. At the end of the incubation period, media was collected, debris was removed by centrifugation, and supernatants were stored at −80°C before assessment of cytokine production by ELISA.

##### 2.3.2.6 Hematoxylin and eosin (H&E) and alcian blue staining

Formalin-fixed, paraffin-embedded colon tissues were sectioned at 4 μm thickness, and sections were stained with H&E or alcian blue as described previously (Wong et al., 2022). Histological impact was evaluated via assessment of intestinal integrity (epithelial architecture), loss of intestinal crypts, ulceration, and infiltration of immune cells/presence of lymphoid follicles. Severity of colitis was quantified using the scoring system previously reported by Hsiung et al. (Hsiung et al., 2014). The scoring system comprises three components, including loss of epithelium, length of crypts, and infiltration of leukocytes, each scored from 0 to 3. A higher total score, which is the summed score of the three components (ranging from 0 to 9), indicates more severe disease. Alcian blue staining was used to assess the secretion of mucin in goblet cells in the colon tissue sections.

##### 2.3.2.7 Immunohistochemical staining

Expression of phospho-IKKα/β, phospho-IκBα, phospho-NF-κB, phospho-STAT3, NLRP3, and CD45 in colon tissues was evaluated via immunohistochemical (IHC) staining. Briefly, paraffin-embedded colon sections (4 μm) were deparaffinized, rehydrated using xylene and alcohol and washed in MilliQ water. The slides were then submitted to antigen retrieval in EDTA pH9.0 buffer for 20 min, followed by blocking with 0.3% hydrogen peroxide for 15 min, and 5% serum with 0.2% triton X-100 for 20 min at room temperature. Slides were incubated with primary antibodies (1:200–1:500) in antibody diluent (Dako) for 1 h at room temperature. HRP-conjugated anti-rabbit or anti-mouse antibodies were added and incubated at room temperature for 30 min. Afterwards, slides were stained with enzyme substrate and counterstained with hematoxylin. Sections were then examined under a bright-field microscope (Nikon eclipse Ni, Tokyo, Japan). Scoring of positively stained cells was carried out independently by two experienced researchers in a blinded fashion; average positive counts as a percentage of control are presented.

### 2.4 Statistical analysis

Data are presented as mean ± SD (cell experiments) or mean ± SEM (animal experiments) from at least three independent experiments. Statistical analysis was performed using Student’s t-test. Values of *P < 0.05, **P < 0.01 and ***P < 0.001 were considered statistically significant.

## 3 Results

### 3.1 LC-MS/MS analysis of SMS

**FIGURE 1:**
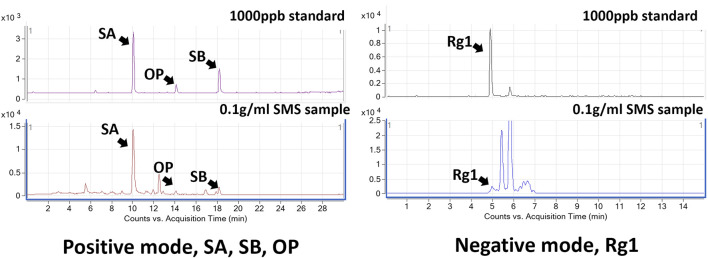
Representative LC-MS/MS chromatograms of SMS. The identified peaks were indicative of Ginsenoside Rg1 (Rg1), Schisandrol A (SA), Schizandrin B (SB), and Ophiopogonin D (OP).

SMS was first characterized using LC-MS/MS analysis to identify the presence of representative chemical constituents. The analysis detected the presence of four key markers: ginsenoside Rg1 (Rg1), schisandrol A (SA), schizandrin B (SB), and ophiopogonin D (OP) (Zheng et al., 2020; Xu et al., 2021). These four compounds are commonly used as chemical markers for the quantification of *Ginseng Radix et Rhizoma*, *Schisandrae chinensis* Fructus, and *Ophiopogonis Radix* respectively. The identity of these compounds was confirmed by comparing of their retention times and peak areas with those of the reference standards. Representative LC-MS/MS fingerprints of the selected chemical markers in SMS and corresponding chemical standards are shown in Figure 1. The quantification of chemical markers in SMS extract is presented in Table 2.

**TABLE 2:** Quantitative assessment of the components in SMS.

Herb	Chemical marker	CAS #	Retention time (minutes)	Amount in SMS extract (μg/g)
*Ginseng Radix et Rhizoma*	Ginsenoside Rg1	41753-43-9	4.97	14.65
*Schisandrae chinensis* Fructus	Schisandrol A	7432-28-2	10.019	411.07
*Schisandrae chinensis* Fructus	Schizandrin B	61281-37-6	12.591	33.75
*Ophiopogonis Radix*	Ophiopogonin D	945619-74-9	14.155	13.34

### 3.2 SMS ameliorated colitis and associated histological changes in DSS-induced mice

**FIGURE 2:**
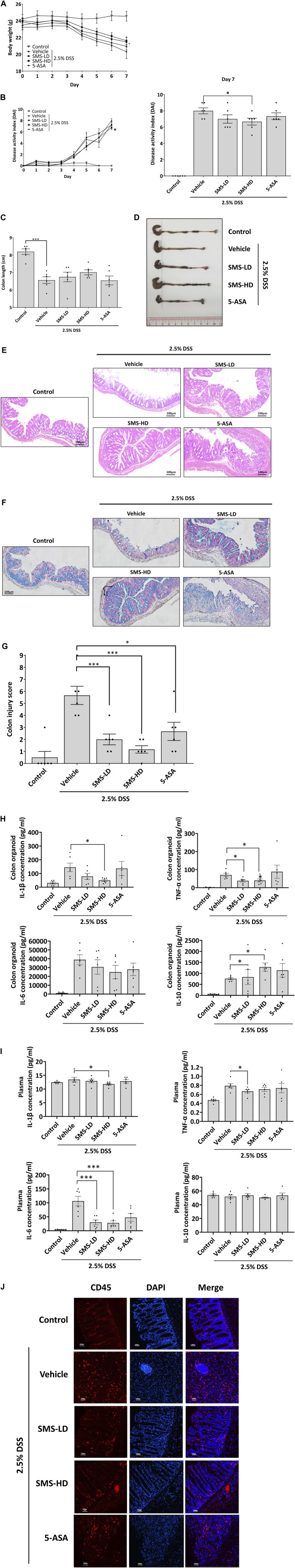
SMS ameliorated DSS-induced colitis in mice. Mice were induced with 2.5% DSS in their drinking water for 7 days. Mice received daily oral administration of vehicle (MilliQ water), SMS-LD (2 g/kg SMS), SMS-HD (4 g/kg SMS), or 5-ASA (200 mg/kg in 0.5% CMC). Control mice received filtered water and MilliQ water treatment. Longitudinal changes in **(A)** body weight, and **(B)** DAI with terminal values also presented. At the end of the experiment, colons were removed, **(C)** lengths measured and **(D)** representative colon images photographed. Histological assessment of colons via **(E)** H&E staining and **(F)** Alcian blue staining. **(G)** Histological scoring of colon damage. Pro-inflammatory cytokine protein levels in **(H)** colon cultures and **(I)** plasma. **(J)** Representative CD45 staining of colon sections. Values shown are means ± SEM of three independent experiments. * P < 0.05, *** P < 0.001 versus the 2.5% DSS with vehicle group.

We next evaluated the *in vivo* anti-inflammatory activity and therapeutic effect of SMS in the well-established DSS-induced acute colitis mouse model. As shown in Figure 2, mice administered with 2.5% DSS via drinking water for 7 days exhibited markedly decreased body weight, increased DAI, shortened colon lengths, and increased pro-inflammatory cytokine levels in colon tissues when compared to control. Treatment of DSS-induced mice with SMS at low (2 g/kg, SMS-LD) or high (4 g/kg, SMS-HD) doses improved body weights (Figure 2A) and food and water consumption (Supplementary Figure S2). SMS-HD elicited significant improvements in DAI, however, improvements in colon length did not reach statistical significance (Figures 2B–D). Furthermore, SMS treatment ameliorated damage in colons induced by DSS. Colons from SMS-treated mice showed reduced epithelial damage and crypt loss when compared with vehicle control or 5-ASA treatment (Figure 2E). Alcian blue staining results also suggested maintenance of mucin secretion in colons of DSS-induced mice after SMS treatment, indicating minimal goblet cell loss (Figure 2F). Histological scoring of colon injury supported the ameliorative effect of SMS on colonic damage (Figure 2G). Further, SMS did not exhibit apparent toxicity in mice; no significant changes in gross morphology of major organs (data not shown) or vital organ weight/body weight coefficients (Supplementary Figure S3) were observed after SMS treatment.

### 3.3 SMS reduced pro-inflammatory cytokine secretion and infiltration of leukocytes in colons of DSS-induced mice

To investigate the potential anti-inflammatory activity of SMS, we examined cytokine secretion in *ex vivo* colon cultures and plasma from DSS-induced mice. As shown in Figure 2H, SMS treatment could decrease the secretion of pro-inflammatory IL-1β and TNF-α from mouse colons, while effects on IL-6 were not significant. Moreover, SMS treatment induced a dose-dependent increase in the production of the anti-inflammatory cytokine, IL-10. In addition, SMS significantly suppressed plasma levels of IL-1β, TNF-α, and IL-6 (Figure 2I). As chemokines released during inflammation can attract leukocytes and result in a positive feedback loop of cytokine production that further induces the inflammatory response, we also examined leukocyte infiltration into colon tissues. Figure 2J shows a substantial increase of CD45-positive staining in the colons of DSS-induced mice receiving vehicle treatment, indicating an increased infiltration of leukocytes. However, SMS treatment suppressed the infiltration of CD45-positive cells.

### 3.4 SMS suppressed pro-inflammatory cytokine secretion and expression in LPS-induced macrophages

**FIGURE 3:**
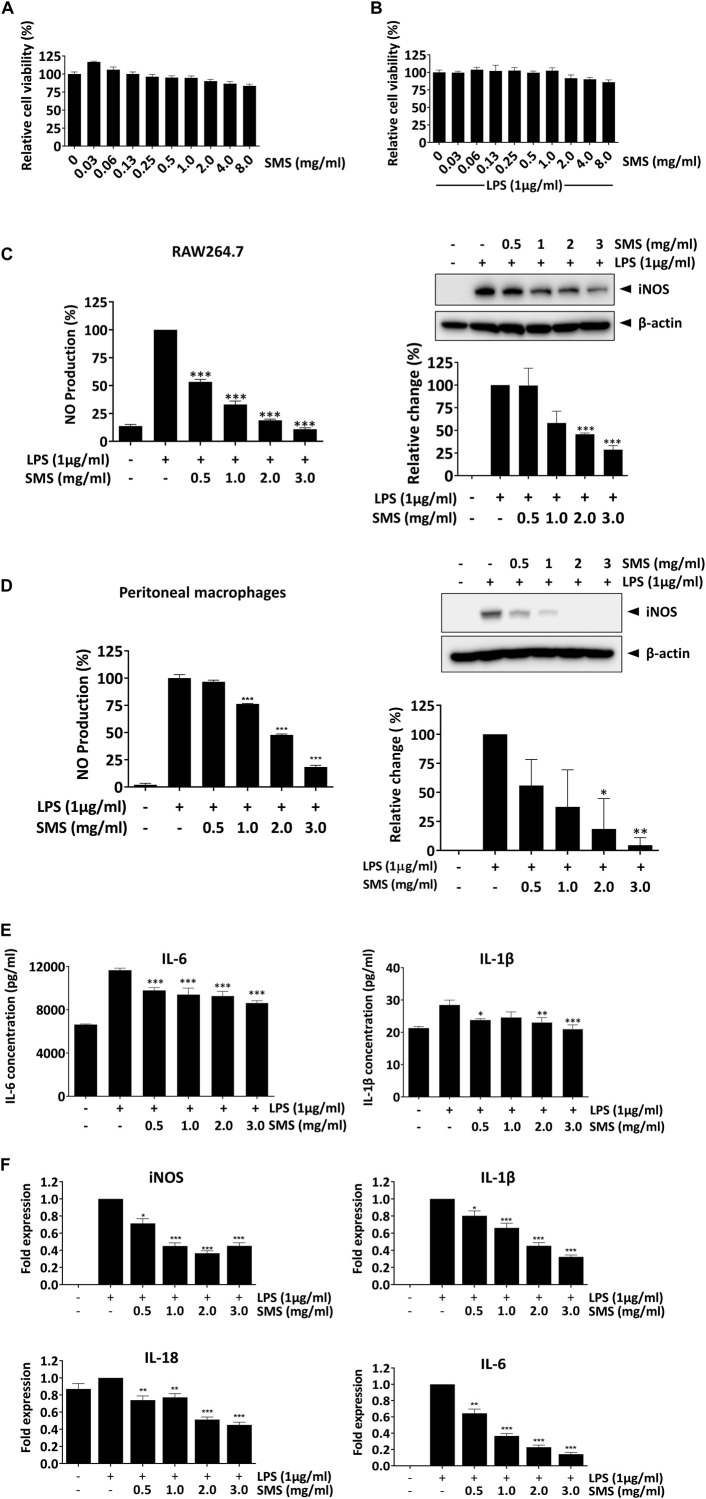
SMS exhibited no cytotoxicity in macrophages and downregulated the expression of LPS-induced pro-inflammatory mediators. RAW264.7 macrophages were treated with SMS at concentrations ranging from 0.03 to 8 mg/mL in the **(A)** absence or **(B)** presence of LPS (1 μg/mL) for 24 h. Cell viability was determined by MTT assay. **(C)** RAW264.7 and **(D)** peritoneal macrophages were pre-treated for 3 h with the indicated concentrations of SMS and further incubated for 24 h with or without LPS (1 μg/mL) before assessment of NO production and iNOS protein expression by Griess assay and Western blotting respectively. Representative immunoblot results and their quantifications are shown. β-actin was used as an internal loading control. **(E)** RAW264.7 macrophages were pre-treated with SMS at the indicated concentrations and further incubated for 24 h with or without LPS (1 μg/mL) before assessment of IL-6 and IL-1β secretion by ELISA. **(F)** Effect of SMS on the mRNA expression of iNOS, IL-1β, IL-18, and IL-6. Data are expressed as means ± SD of three independent experiments. *P < 0.05, **P < 0.01, ***P < 0.001 compared to LPS-induced control.

To elucidate the anti-inflammatory mechanism of SMS, we studied the effects of SMS in LPS-induced RAW264.7 macrophages. First, the cytotoxicity of SMS was evaluated in macrophages with or without LPS induction. SMS exhibited no cytotoxicity at all tested concentrations (Figures 3A, B), and doses of 0.5–3 mg/mL were selected for downstream studies. Next, we investigated the anti-inflammatory activity of SMS by way of NO production and expression of its regulatory enzyme iNOS in both LPS-induced RAW264.7 macrophages and primary peritoneal macrophages. Treatment with SMS could significantly decrease LPS-induced NO production and iNOS expression in a dose-dependent manner. Notably, 3 mg/mL SMS significantly suppressed the induction of NO and iNOS by about 90% when compared to control in both cells (Figures 3C, D). We also examined the protein and gene expression levels of pro-inflammatory cytokines in LPS-induced RAW264.7 macrophages after SMS treatment. Figures 3E, F show that SMS treatment suppressed the secretion and mRNA expression of IL-6 and IL-1β. Furthermore, SMS could suppress iNOS and IL-18 mRNA levels in a dose-dependent manner (Figure 3F). These results demonstrated the suppressive effect of SMS on LPS-induced macrophages.

### 3.5 SMS suppressed NF-κB signaling in LPS-induced macrophages

**FIGURE 4:**
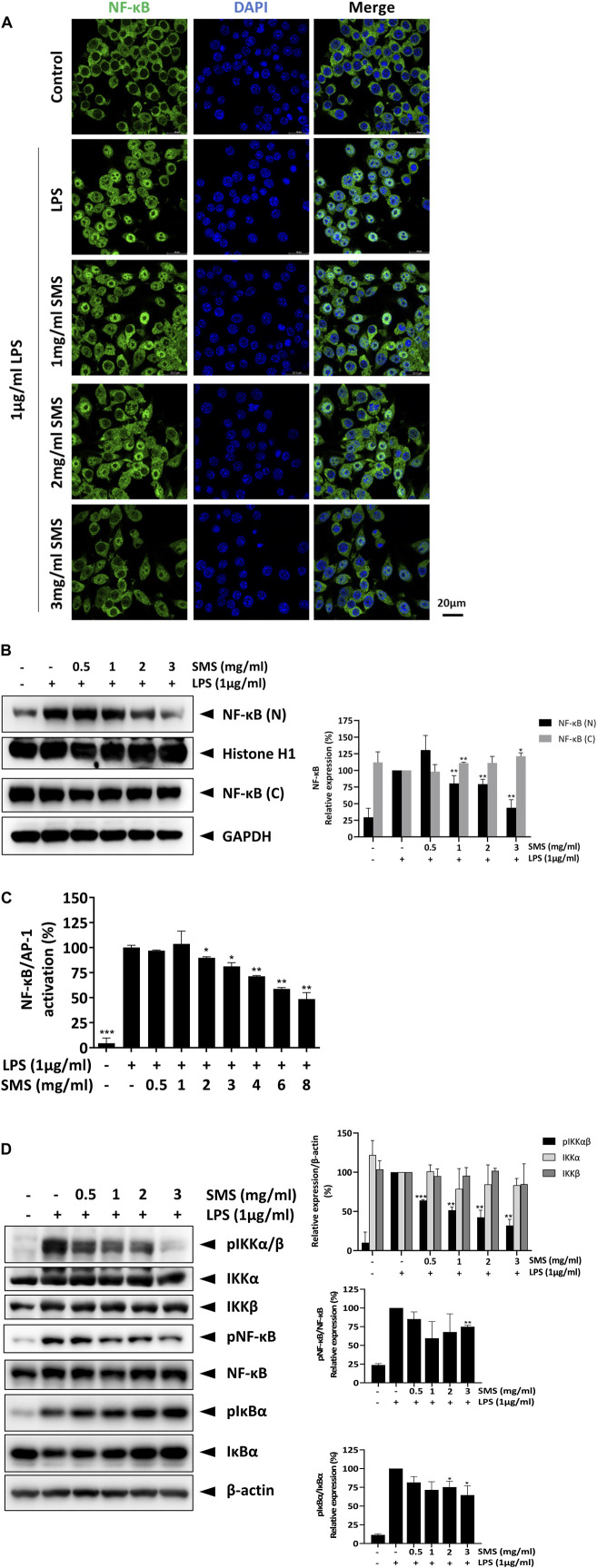
SMS inhibited the activation of NF-κB signaling. RAW264.7 macrophages were pre-treated with SMS followed by co-treatment with LPS. The effect of SMS on the activation of NF-κB signaling was evaluated by assessment of **(A)** translocation of NF-κB using immunofluorescence staining, **(B)** nuclear (N) and cytosolic (C) NF-κB expression, and **(C)** J774 Dual NF-κB reporter cell activity. RAW264.7 macrophages were pre-treated with the indicated concentration of SMS for 3 h and further incubated for 15 min with or without LPS (1 μg/mL) before Western blot assessment of **(D)** protein phosphorylation levels of IKKα, IKKβ, NF-κB, and IκBα in total cell lysates. Histone H1, GAPDH, and β-actin were used as internal loading controls. Representative immunoblot results and their quantifications are shown. Data are expressed as means ± SD of three independent experiments. *P < 0.05, **P < 0.01, ***P < 0.001 compared to LPS-induced control.

Next, we investigated the molecular signaling involved in the inhibitory effects of SMS. As the transcription factor NF-κB serves as a pivotal mediator of inflammatory responses, we examined the effect of SMS on NF-κB signaling. As shown in Figure 4A, SMS treatment dose-dependently suppressed nuclear translocation of NF-κB/p65 when compared with LPS control. Assessment of NF-κB in nuclear and cytoplasmic fractions showed that SMS could dose-dependently suppress NF-κB expression in the nucleus, further supporting the effect of SMS on NF-κB translocation (Figure 4B). Results showed that SMS could also significantly suppress NF-κB/p65-mediated transcriptional activation of LPS-induced J774-Dual reporter cells (Figure 4C). We then studied the effect of SMS on the protein expression of molecules involved in NF-κB signaling, including NF-κB and its upstream regulators, IκB-α and IKKα/β. When compared to LPS control, SMS could downregulate the expression of phosphorylated -IKKα/β, -IκBα, and -NF-κB in a dose-dependent manner (Figure 4D). Altogether, these results suggested that the anti-inflammatory activity of SMS was potentially mediated through inhibition of the NF-κB signaling pathway.

### 3.6 SMS suppressed MAPK and STAT3 signaling in LPS-induced macrophages

**FIGURE 5:**
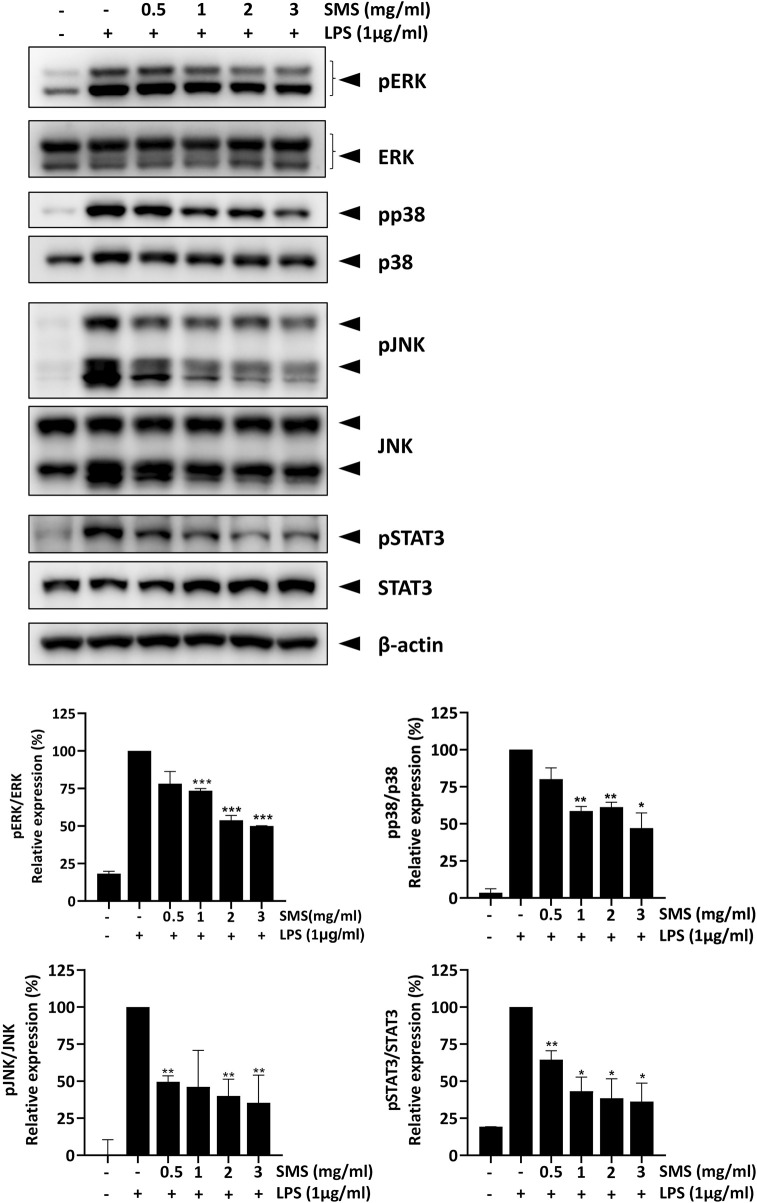
SMS inhibited activation of MAPK and STAT3 signaling. RAW264.7 macrophages were pre-treated with the indicated concentrations of SMS for 3 h and further incubated for 15 min with or without LPS (1 μg/mL). Effect of SMS on the protein levels of phosphorylated ERK, p38, JNK, and STAT3 were examined using Western blot. β-actin was used as a loading control. Representative immunoblot results and their quantifications are shown. Data are expressed as means ± SD of three independent experiments. *P < 0.05, **P < 0.01, ***P < 0.001 compared to LPS-induced control.

Pro-inflammatory cytokines such as IL-1β, IL-6, and TNF-α can trigger the activation of MAP kinase and STAT3 signaling and form a positive loop that promotes and sustains the inflammatory response. These signaling pathways have been demonstrated to be closely interlinked with NF-κB signaling and are activated in IBD patients (Yu et al., 2020; Bouwman et al., 2022). Therefore, we investigated the effect of SMS on key MAP and STAT3 kinases. As shown in Figure 5, SMS treatment could significantly and dose-dependently inhibit the phosphorylation of ERK, JNK, p38, and STAT3 in LPS-induced macrophages, suggesting SMS also exhibited suppressive effects on MAP and STAT3 signaling.

### 3.7 SMS suppressed NLRP3 activation *in vitro*

As the NLRP3 inflammasome has emerged as a crucial regulator of intestinal homeostasis and has been widely associated with the pathogenesis and progression of IBD (Bauer et al., 2010; Liu et al., 2017), and studies have shown that inhibition of the NLRP3 inflammasome is effective for alleviation of IBD (Weber et al., 2023), we investigated the potential activity of SMS on NLRP3 inflammasome signaling.

**FIGURE 6:**
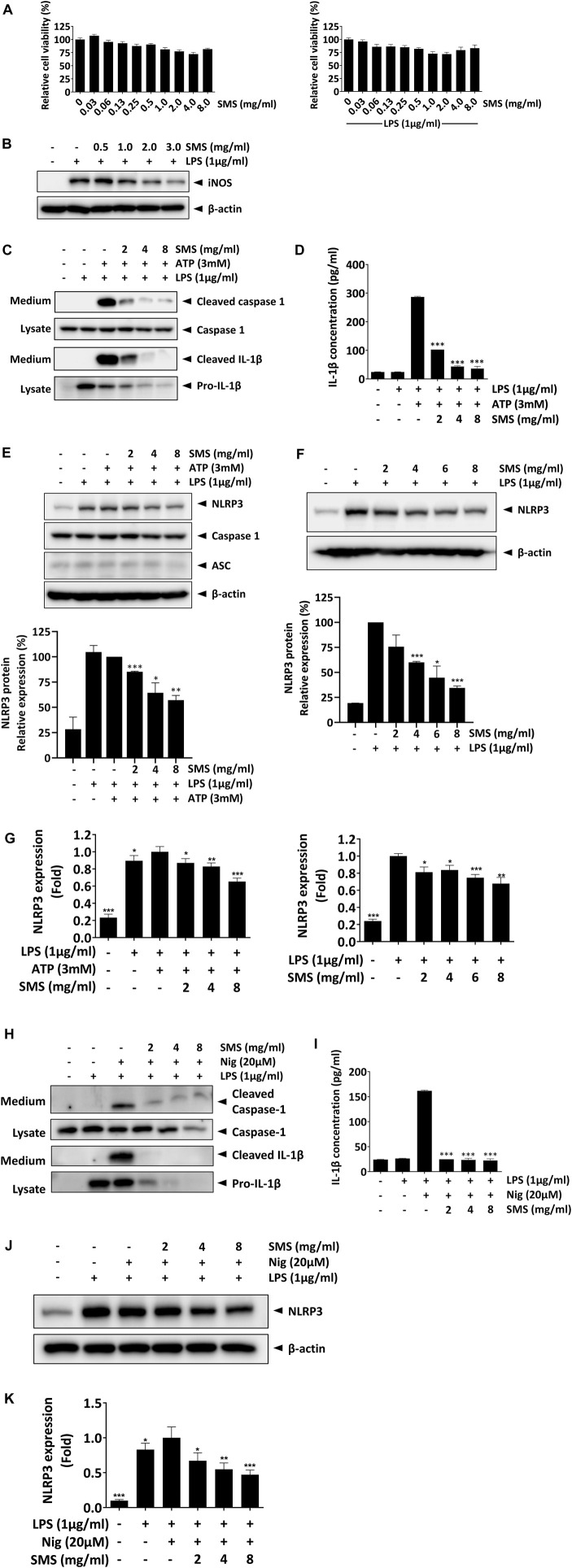
SMS suppressed NLRP3 inflammasome signaling in J774.1 macrophages. Inhibitory activity of SMS on NLRP3 inflammasome signaling was evaluated in J774A.1 macrophages. **(A)** Effect of SMS on J774A.1 cell viability with or without LPS induction. **(B)** Effect of SMS on LPS-induced iNOS expression. LPS-primed J774A.1 macrophages were stimulated with or without 3 mM ATP or 20 μM nigericin for 30 min in the presence or absence of the indicated concentrations of SMS. Under induction by LPS and ATP, **(C)** effect of SMS on the secretion of cleaved caspase 1 and mature IL-1β, assessed via Western blot, **(D)** effect of SMS on the secretion of mature IL-1β, assessed via ELISA, and **(E)** effect of SMS on the protein expression of NLRP3, caspase 1, and ASC. **(F)** Effect of SMS on the protein expression of NLRP3 under LPS induction only. **(G)** Effect of SMS on the mRNA expression of NLRP3 in LPS-primed macrophages with or without ATP induction. Effect of SMS on the LPS and nigericin-induced secretion of cleaved caspase 1 and mature IL-1β was assessed via **(H)** Western blot or **(I)** ELISA. Effect of SMS on the **(J)** protein and **(K)** mRNA expression of NLRP3 in LPS and nigericin-induced J774A.1 cells. Data are expressed as means ± SD of three independent experiments. *p < 0.05, **p < 0.01, ***p < 0.001 compared to induced control.

We first evaluated the cytotoxicity of SMS in J774A.1 macrophages. J774A.1 cells were used for this part of the study as RAW264.7 cells lack expression of ASC, a core component of the NLRP3 inflammasome, and is thus deficient in activation of the pathway (Pelegrin et al., 2008). In concordance with the results in RAW264.7 cells, no cytotoxicity was observed among the tested doses (Figure 6A). Furthermore, SMS could significantly suppress iNOS expression in J774A.1 macrophages, confirming that SMS exhibited anti-inflammatory activity in this cell line (Figure 6B). We then examined the effect of SMS on expression of key protein markers of the NLRP3 signaling pathway. Complete activation of the NLRP3 inflammasome requires both a priming signal from LPS and an activation signal from a second stimulus (e.g., ATP or nigericin). Upon NLRP3 inflammasome activation, recruited caspase 1 is cleaved to generate a complex of p33/p10, which remains bound to the inflammasome. Further processing of p33 will then release p20/p10 (Boucher et al., 2018; Swanson et al., 2019). As p20/p10 is unstable in cells, it is difficult to detect cellular caspase 1 expression and thus we examined cleaved caspase 1 in culture media (Hughes et al., 2019; Bai et al., 2022). In culture media of LPS-primed J774A.1 macrophages treated with ATP, SMS treatment could significantly reduce cleaved-caspase 1 expression and IL-1β maturation, as well as IL-1β secretion (Figures 6C, D). These results indicated that SMS could inhibit NLRP3 inflammasome-induced caspase-1 cleavage and subsequent IL-1β secretion.

The NLRP3 inflammasome is a multimeric complex consisting of NLRP3, ASC, and caspase 1; we postulated that SMS might mediate the protein expression of these molecules, resulting in its suppressive effect on the NLRP3 inflammasome. As shown in Figure 6E, while the expression of ASC and caspase-1 were unaffected, SMS treatment significantly inhibited NLRP3 expression in a dose-dependent manner. As expression of NLRP3 can be regulated by the LPS priming signal during inflammasome activation, we evaluated the inhibitory effect of SMS on NLRP3 in LPS-primed macrophages. As shown in Figure 6F, and consistent with our previous results, SMS dose-dependently inhibited NLRP3 protein expression. Further, we assessed the effect of SMS on gene expression of NLRP3 in J774A.1 macrophages under induction by LPS alone or together with ATP. Results showed that SMS could significantly and dose-dependently reduce the mRNA levels of NLRP3 under both conditions (Figure 6G). Another commonly used NLRP3-specific activator, nigericin, was used in parallel to confirm the inhibitory effect of SMS on the activation of NLRP3 signalling, and similar inhibitory effects were observed (Figure 6H–K). Taken together, these data indicated that SMS could inhibit activation of the NLRP3 inflammasome via suppression of NLRP3 expression.

### 3.8 SMS downregulated NF-κB and NLRP3 inflammasome signaling in colon tissues of DSS-induced mice

**FIGURE 7:**
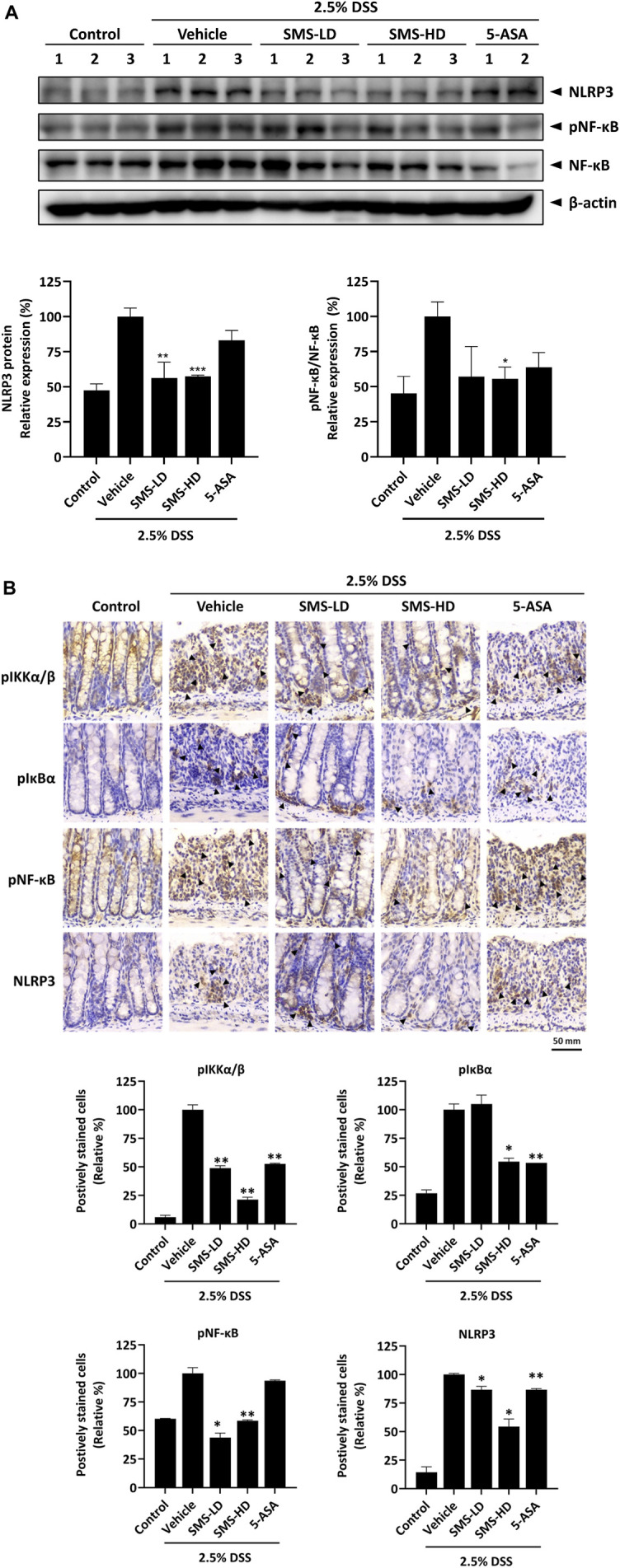
SMS inhibited NF-κB and NLRP3 signaling in DSS-induced mice. Inhibitory activity of SMS on NF-κB and NLRP3 pro-inflammatory signaling was examined in colon tissues of DSS-induced mice. **(A)** Protein expression of phosphorylated NF-κB and NLRP3. β-actin was used as a loading control. Representative immunoblot results and their quantifications are shown. **(B)** Immunohistochemical staining of colon tissues against key members of the NF-κB and NLRP3 signaling. Positively stained cells were counted independently by two experienced researchers in a blinded manner and their quantifications are shown. *p < 0.05, **p < 0.01 compared to induced control.

To validate the inhibitory effects of SMS on NF-κB and NLRP3 inflammasome signaling *in vivo*, we examined their expression in DSS-induced mice. As shown in Figure 7A, the expression of NLRP3 and phosphorylated NF-κB in colon tissues of DSS-induced mice was significantly reduced after SMS-LD or SMS-HD treatment. This suppressive effect was further confirmed by immunohistochemical analysis of mouse colon tissues, where SMS exerted significant inhibition on phosphorylated -IKKα/β, -IκB-α, -NF-κB, and NLRP3 (Figure 7B). In conclusion, our results demonstrated a notable suppressive effect of SMS on NF-κB and NLRP3 inflammasome signaling.

## 4 Discussion

IBD is a recurring lifelong inflammatory disease, currently without curative treatment. Common therapies for IBD patients include drug treatments such as 5-ASA and corticosteroids (Kayal and Shah, 2019). However adverse side effects of these treatments on gastrointestinal and haematological systems often hinder their usage (Voss, 2019). In addition to adverse effects, 5-ASA, corticosteroids, and immunotherapies such as antibiotics and biologics often show a lack of long-term efficacy in maintenance of IBD remission (Kayal and Shah, 2019). As a result, the discovery of safe and effective therapeutic agents against IBD is of utmost importance. SMS is a Chinese traditional herbal formula that has been shown to exhibit anti-inflammatory potential in recent studies. Therefore, we investigated the anti-inflammatory effects and potential mechanisms of SMS, as well as its potential as a therapeutic agent against IBD.

Previous clinical studies have demonstrated that SMS could improve clinical outcomes in patients with acute myocardial infarction, coronary heart disease, and heart failure. In addition, SMS could also improve pulmonary gas exchange in patients after tourniquet-induced ischemia-reperfusion, and respiratory function in patients with chronic obstructive pulmonary disease (Leong and Ko, 2018). In this study, we showed for the first time the therapeutic potential of SMS in IBD. SMS treatment could ameliorate disease severity, improve intestinal histology, reduce colonic immune cell infiltration, and inhibit pro-inflammatory mediator expression and signaling.

The three components of SMS, *Ginseng Radix et Rhizoma*, *Ophiopogonis Radix*, and *Schisandrae chinensis* Fructus, have each been reported to exhibit anti-inflammatory activities in previous studies. Ginsenoside Re (Lee et al., 2012), ginsenoside Rb1 (Zhang et al., 2015), and ginsenoside compound K (Li et al., 2014) in *Ginseng Radix et Rhizoma* have been reported to exhibit modulatory effects on NF-κB signaling. In addition, ginsenoside Rd can also suppress activation of the NLRP3 inflammasome (Liu et al., 2018). In *Ophiopogonis Radix*, methylophiopogonanone A, methylophiopogonanone B, ophiopogonanone A, and ophiopogonin B have been shown to suppress the expression of pro-inflammatory cytokines (Kitahiro et al., 2018). Furthermore, polysaccharides isolated from *Schisandrae chinensis* Fructus were shown to exhibit inhibitory effects in colitis mice via modulation of the gut microbiota (Su et al., 2020). Thus, each of the three herbal components in SMS may exhibit anti-inflammatory effects via different mechanisms and when used as a formula, could potentially contribute to a superior therapeutic efficacy when compared to single herbs or commonly used IBD therapeutics such as 5-ASA.

iNOS is the enzyme responsible for catalysing the production of nitric oxide (NO). Under the pro-inflammatory response, iNOS is predominantly expressed at the site of inflammation, leading to increased synthesis of NO. Studies have shown that elevated levels of iNOS and NO were detected in colon biopsies from IBD patients, indicating the relationship between iNOS-dependent NO production and disease development in IBD (Gochman et al., 2012). Previous studies have demonstrated that SMS treatment could ameliorate cerebral iNOS reactivity and NO production, and reduce serum levels of TNFα, IL-1β, and IL-6 in heat stroke rats (Wang et al., 2005). Our results demonstrated the potent suppressive effects of SMS on iNOS and NO in LPS-induced macrophages, supporting the anti-inflammatory efficacy of SMS.

Furthermore, iNOS expression is tightly regulated by several signaling cascades, including the NF-κB, MAPK, and STAT signaling pathways. Upon detection of bacterial components such as LPS by toll-like receptor 4 (TLR4), macrophages are activated into a pro-inflammatory status. The dissociation of IκB from NF-κB enables its translocation into the nucleus, where it activates gene transcription of pro-inflammatory cytokines (Li et al., 2002). Moreover, activation of TLR4 also leads to the phosphorylation of MAPK and STAT3. Studies have indicated that the mRNA and protein expression of NF-κB, MAPK, and STAT3 were upregulated in biopsies of IBD patients, especially in colonic macrophages, together with an increase in pro-inflammatory cytokines including TNFα, IL-1β, and IL-6 (Papoutsopoulou et al., 2019). In a previous study, SMS was demonstrated to exhibit a suppressive effect on cardiac IL-6 levels in doxorubicin-induced cardiac toxicity rats (Ma et al., 2016), suggesting its anti-inflammatory potential. In our study, results demonstrated that SMS treatment suppressed the expression levels of NF-κB, IKKα/β, and IκBα in the colons of DSS-induced mice, along with a reduction in pro-inflammatory cytokine release from colon organoids. In LPS-induced macrophages, SMS reduced the nuclear translocation of NF-κB, resulting in suppression of the promoter activity of NF-κB/AP-1, and leading to an overall suppression of NF-κB activity. We have thus demonstrated the inhibitory effect of SMS on the NF-κB, MAPK, and STAT3 signaling pathways underlying its anti-inflammatory activity.

In addition to the above signaling pathways, studies have also suggested a critical role for the NLRP3 inflammasome signaling pathway in IBD pathogenesis and progression. Activation of the NLRP3 inflammasome triggers production of pro-inflammatory cytokines, of which IL-1β can further induce the inflammatory response and lead to a sustained activation of macrophages, enhancing inflammation in the gut and aggravating colonic damage (Kelley et al., 2019; Chen et al., 2021). NLRP3 transcription was found to be highly upregulated in IBD patients, and aberrant activation of the NLRP3 inflammasome has also been observed and associated with an increased risk of IBD pathogenesis (Perera et al., 2018; Chen et al., 2021). In addition, increased IL-1β levels were seen in IBD patients (Lazaridis et al., 2017). Meanwhile, studies have demonstrated that suppression of NLRP3 inflammasome activation led to inhibition of pro-inflammatory cytokine production and anti-inflammatory effects in macrophages (Seok et al., 2021). Therefore, targeting NLRP3 signaling represents a promising therapeutic approach for the treatment of IBD. In our *in vitro* studies, we evaluated the inhibitory effect of SMS on NLRP3 signaling in J774A.1 macrophages induced by LPS and either ATP or nigericin. ATP acts as an agonist which indirectly regulates NLRP3 activity via stimulation of P2X_7_ nonselective K^+^ or Ca^2+^ channel receptors, while nigericin is described as NLRP3-specific activator, acting as a potassium ionophore by facilitating H^+^/K^+^ anti-port across cell membranes and thereby activating NLRP3 via potassium efflux (Katsnelson et al., 2015). As demonstrated in our *in vitro* results, SMS treatment significantly reduced the transcription and protein expression of NLRP3, and significantly inhibited the levels of cleaved caspase 1 and mature IL-1β in ATP- or nigericin-induced macrophages. In addition, immunohistochemical evaluation also indicated a decrease in NLRP3 expression in colon sections of DSS-induced mice, suggesting a potential role for SMS in the treatment of IBD through suppression of NLRP3.

To conclude, the potent anti-inflammatory effects of SMS were demonstrated in both *in vivo* and *in vitro* models. SMS ameliorated colonic damage in DSS-induced acute colitis mice through suppression of pro-inflammatory cytokine production via inhibition of NF-κB and NLRP3 inflammasome signaling cascades. Altogether, this study provides insight into the anti-inflammatory effects of SMS and the potential mechanisms involved, highlighting the promise for further development of SMS as a therapeutic agent against IBD.

## Data Availability

The original contributions presented in the study are included in the article/Supplementary Material, further inquiries can be directed to the corresponding authors.

## Abbreviations

5‐ASA, 5‐aminosalicylic acid; DAI, Disease activity index; DMEM, Dulbecco's modified eagle medium; DSS, Dextran sulfate sodium; ELISA, Enzyme-linked immunosorbent assay; FBS, Fetal bovine serum; H&E, Hematoxylin and Eosin; IBD, Inflammatory bowel disease; IHC, Immunohistochemistry; LPS, Lipopolysaccharide; MTT, 3‐[4, 5-dimethylthiazol‐2‐yl]‐2, 5‐diphenyltetrazoliumbromide; NO, Nitric oxide; qPCR, Quantitative polymerase chain reaction; SEAP, Secreted embryonic alkaline phosphatase; SMS, Sheng Mai San; TCM, Traditional Chinese medicine; TLR4, Toll-like receptor 4.
