# Supplementary material for: Therapeutic effect of Sheng Mai San, a traditional Chinese medicine formula, on inflammatory bowel disease via inhibition of NF-κB and NLRP3 inflammasome signaling
Source: Front Pharmacol. 2024 Aug 2;15:1426803. doi: 10.3389/fphar.2024.1426803 (PMC11327010; doi:10.3389/fphar.2024.1426803)
Supplement: Supplementary file 1 [file DataSheet1.docx]

Supplementary Material

**
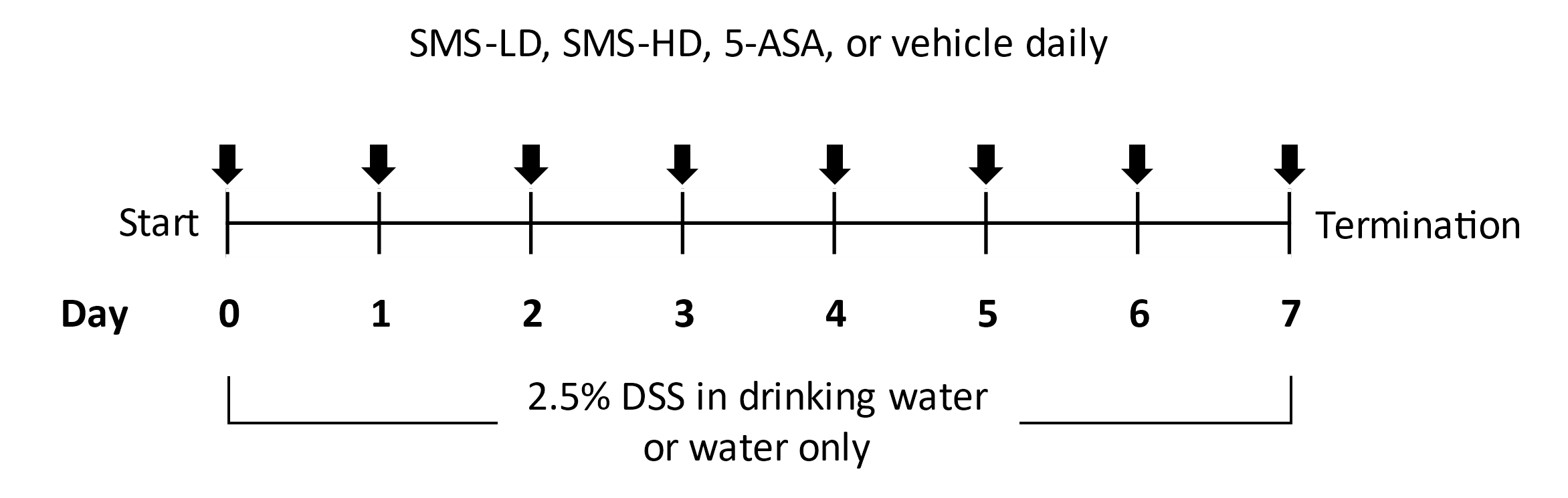
**

**Supplementary Figure 1.** Experimental treatment scheme of DSS-induced acute colitis mice.

**
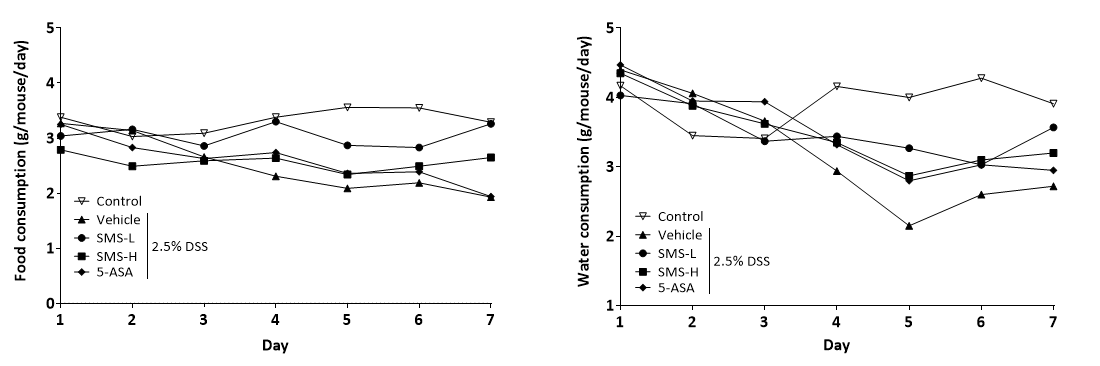
**

**Supplementary Figure 2.** SMS improved food and water consumption of DSS-induced acute colitis mice.

**
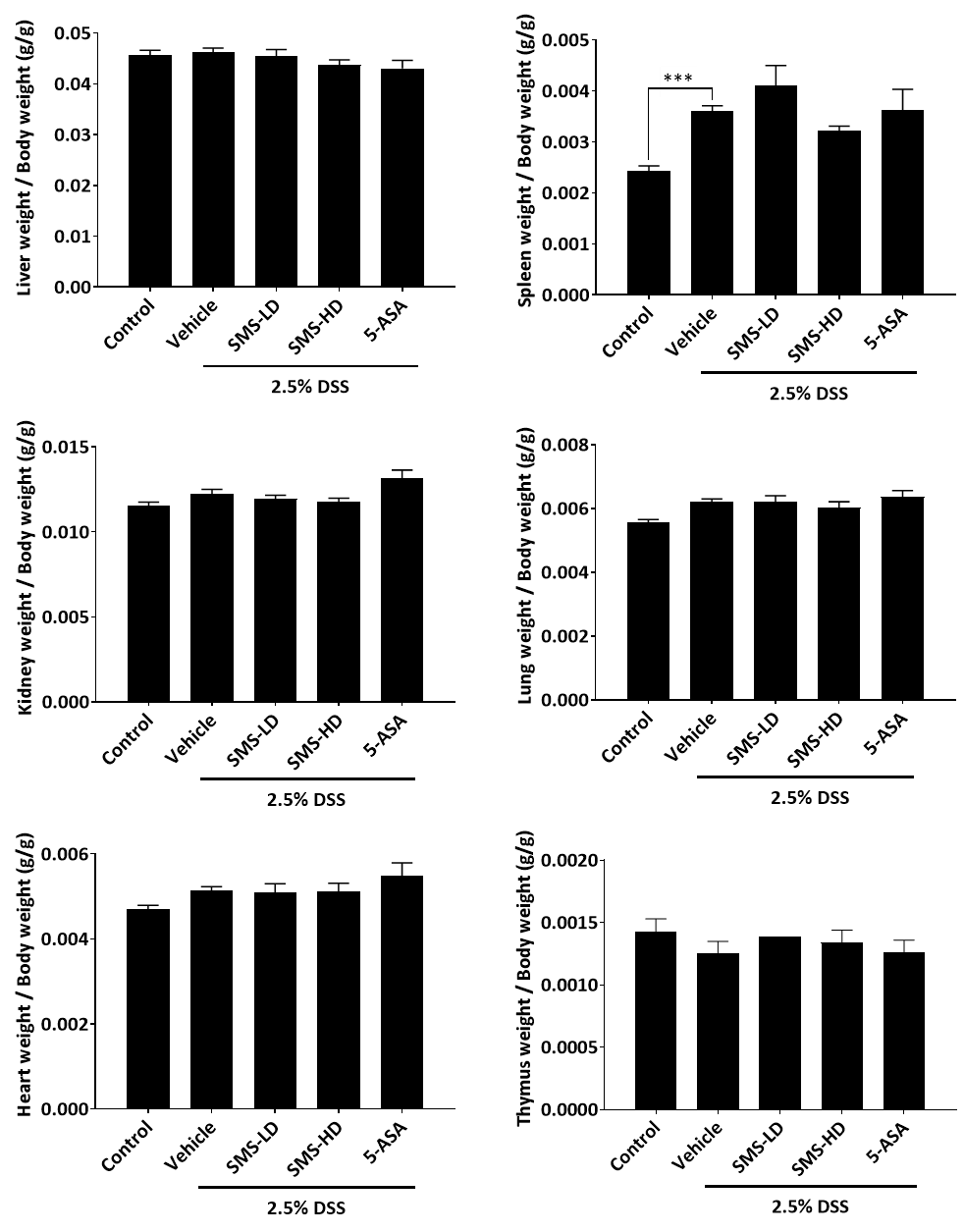
**

**Supplementary Figure 3.** SMS did not affect vital organ/body weight coefficients of DSS-induced acute colitis mice.

**Supplementary Table 1 – qPCR primers**

| **Gene** | **Forward primer 5’ - 3’** | **Reverse primer 5’ - 3’** |
| --- | --- | --- |
| iNOS | GCATTGGAAGTGAAGCGTTTC | GGCAGCCTGTGAGACCTTTG |
| IL-6 | CAGAAGGAGTGGCTAAGGACCA | ACGCACTAGGTTTGCCGAGTAG |
| IL-1β | TGGGGAACTCTGCAGACTCAAAC | GGAGAACCAAGCAACGACAAAATA |
| TNF-α | GGTGAGGAGCACGTAGTCGG | TCCCAGGTTCTCTTCAAGGGA |
| IL-10 | AACATACTGCTAACCGACTCCT | CTGCCTTGCTCTTATTTTCACA |
| NLRP3 | TCGCAGCAAAGATCCACACAG | ATTACCCGCCCGAGAAAGG |
| β-actin | GACAGGATGCAGAAGGAGATTACT | TGATCCACATCTGCTGGAAGGT |
